# Ancient Recombination Events between Human Herpes Simplex Viruses

**DOI:** 10.1093/molbev/msx113

**Published:** 2017-03-28

**Authors:** Sonia Burrel, David Boutolleau, Diane Ryu, Henri Agut, Kevin Merkel, Fabian H. Leendertz, Sébastien Calvignac-Spencer

**Affiliations:** 1 National Reference Centre for Herpesviruses, Paris, France; 2 AP-HP, University Hospital La Pitié-Salpêtrière – Charles Foix, Virology Department and Sorbonne Universités, UPMC Univ Paris 06, CR7, CIMI, INSERM U1135, Paris, France; 3 Robert Koch Institut, Berlin, Germany

**Keywords:** human herpes simplex virus, phylogenomics, recombination

## Abstract

Herpes simplex viruses 1 and 2 (HSV-1 and HSV-2) are seen as close relatives but also unambiguously considered as evolutionary independent units. Here, we sequenced the genomes of 18 HSV-2 isolates characterized by divergent UL30 gene sequences to further elucidate the evolutionary history of this virus. Surprisingly, genome-wide recombination analyses showed that all HSV-2 genomes sequenced to date contain HSV-1 fragments. Using phylogenomic analyses, we could also show that two main HSV-2 lineages exist. One lineage is mostly restricted to subSaharan Africa whereas the other has reached a global distribution. Interestingly, only the worldwide lineage is characterized by ancient recombination events with HSV-1. Our findings highlight the complexity of HSV-2 evolution, a virus of putative zoonotic origin which later recombined with its human-adapted relative. They also suggest that coinfections with HSV-1 and 2 may have genomic and potentially functional consequences and should therefore be monitored more closely.

## Introduction

Human herpes simplex virus 1 and 2 (HSV-1 and 2; species *Human alphaherpesvirus 1* and *2*, genus *Simplexvirus*, subfamily *Alphaherpesvirus*, family *Herpesviridae*, order *Herpesvirales*) are closely related pathogens that usually cause recurrent mucosal lesions but are also, more rarely, responsible of severe diseases like neonatal morbidity and meningo-encephalitis. HSV-1 is essentially transmitted by oral-oral contacts and is responsible for oro-facial herpes whereas HSV-2 is primarily sexually transmitted and causes genital herpes. However, HSV-1 can be transmitted through oral sex and consequently cause genital herpes. Infection with HSV-1 is extremely common and affects 67% of the worldwide population (Looker, Magaret, May, et al. 2015). Infection with HSV-2 is less frequent but still reaches a global prevalence of 11% (Looker, Magaret, Turner, et al. 2015). Important regional variations in prevalence exist for both viruses, with the highest prevalence observed in Africa (87% and 31% for HSV-1 and HSV-2, respectively; Looker, Magaret, May, et al. 2015; Looker, Magaret, Turner, et al. 2015).

HSV-1 and HSV-2 show low overall genomic variability. HSV-1, however, has accumulated more variation than HSV-2 (maximum overall divergence 1.1% vs. 0.4%; Kolb et al. 2011, 2013, 2015; Szpara et al. 2014; Newman et al. 2015). Most HSV-2 open reading frames (ORF) exhibit little (if any) variability. Yet we recently discovered a HSV-2 variant (HSV-2v) characterized by unusually divergent UL30 gene sequences (maximum divergence of 2.4%; Burrel et al. 2013, 2015). Although HSV-2 genomic diversity is only weakly, if at all, geographically structured (Kolb et al. 2015; Newman et al. 2015), this new variant was also exceptional in that it was mainly recovered from subSaharan African individuals. To clarify the evolutionary history of HSV-2v, we generated nearly complete genomes from 18 isolates originating in distinct patients (table 1), which we compared with other HSV-2 and the closest alphaherpesviruses.

**Table 1:** Characteristics of Patients Infected by HSV-2v.

Patient	Gender	Age (years)	Country of Origin	HIV Infection	Site of Infection
1	M	48	Guinea	HIV-1	Genital
2	F	36	Niger	HIV-1	Buttock
3	F	38	Nigeria	HIV-1	Anal
4	F	30	Côte d’Ivoire	HIV-1	Genital
5	M	5	Unspecified (African)	No	Buttock
6	M	52	Equatorial Guinea	HIV-1	Buttock
7	F	22	Mali	No	Genital
8	M	44	Mali	No	Genital
9	M	46	Côte d’Ivoire	HIV-2	Genital
10	M	55	Democratic Republic of the Congo	No	Genital
11	F	29	Côte d’Ivoire	HIV-2	Buttock
12	F	61	Côte d’Ivoire	HIV-1	Genital
13	F	32	Democratic Republic of the Congo	No	Genital
14	M	63	Unspecified (Caucasian)	HIV-1	Buttock
15	M	NA	Martinique (West Indies)	Unknown	Genital
16	F	NA	Unspecified (African)	Unknown	Genital
17	M	65	Burundi	No	Genital
18	M	65	Unspecified (African)	Unknown	Genital

Note.—NA, not available; M, male; F, female.

## Results

After quality filtering, mapping and deduplication, we determined the sequence of 75% of the genomes (28–90%; excluding the terminal repeats of the long and short regions, TRL and TRS), from which we identified 10–72 coding sequences per genome (74 coding sequences in the reference genome; table 2). We used consensus sequences to generate a whole genome alignment that also included the sequences from all georeferenced simplexvirus genomes determined from hominine hosts, i.e., HSV-1, HSV-2, and the only sequence derived from a chimpanzee (chimpanzee alpha-1 herpesvirus, ChHV; Severini et al. 2013), as well as the sequence of the cercopithecine herpesvirus 2 (Tyler et al. 2005) (supplementary table S1, Supplementary Material online, p. 2–5).

**Table 2:** Sequencing Results.

Isolate	Raw Reads	Mapped Reads (after trimming and deduplicating)	Unmapped Reads (after trimming and deduplicating)	Proportion of On-Target Unique Reads	Average Unique Coverage Depth	1× Genome Coverage^a^	>20× Genome Coverage and >95% Base Call Agreement^b^	Extracted Open Reading Frames
1	723314	168395	60288	0.74	167	0.96	0.81	66
2	455134	185657	42482	0.81	178	0.96	0.83	67
3	885188	187518	137516	0.58	175	0.95	0.81	67
4	63439	25050	1960	0.93	19	0.84	0.30	10
5	452452	200550	34774	0.85	170	0.97	0.84	67
6	215351	101850	53884	0.65	98	0.95	0.76	64
7	1522881	358859	206436	0.63	392	0.97	0.90	71
8	200828	100949	16072	0.86	107	0.95	0.78	64
9	359998	136117	95664	0.59	126	0.96	0.80	66
10	854637	223955	27414	0.89	219	0.95	0.82	66
11	38509	14248	11284	0.56	16	0.84	0.28	13
12	284066	87582	102070	0.46	85	0.94	0.72	60
13	246454	111929	24338	0.82	108	0.95	0.77	63
14	513116	142870	78814	0.64	135	0.95	0.75	60
15	1789464	530287	113636	0.82	507	0.98	0.86	72
16	191070	79210	42396	0.65	86	0.94	0.74	62
17	818411	359469	76382	0.82	377	0.98	0.89	70
18	294052	115158	4430	0.96	127	0.95	0.78	63

a Fraction of the genome covered by at least one read.

b Fraction of the genome sequence determined when only positions covered by more than 20 reads and where 95% of the reads agreed on the base call are validated.

### Recombination Analysis

**Fig. 1.**
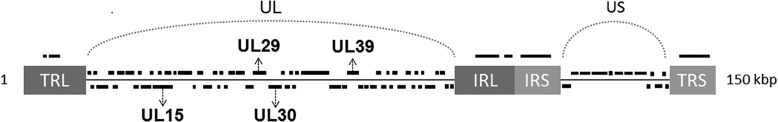
Map of the HSV-2 genome. The HSV genome is a linear double-stranded DNA of around 150 kbp that encodes at least 74 genes (black rectangles). It is composed of two covalently linked segments named as unique long (UL) and unique short (US) sequences (heavy solid lines). Each segment is bracketed by the terminal and internal inverted repeats sequences TRL-IRL and IRS-TRS (gray boxes). kbp: kilo base pairs.

**Fig. 2.**
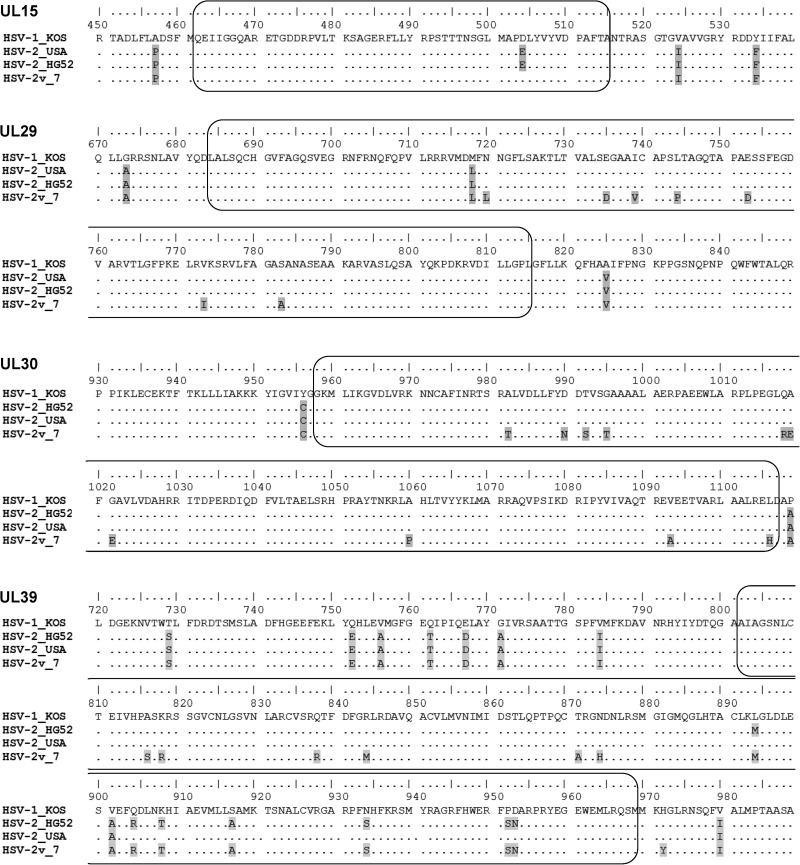
Amino acid alignments of recombinant fragments. These four alignments are focused on the recombinant regions and only cover parts of the respective coding sequences. Reference strains: KOS [HSV-1] named HSV-1_KOS and HG52 [HSV-2] named HSV-2_HG52, GenBank accession numbers KT899744 and NC001798, respectively. Clinical strains: HSV-2v isolate number 7 (from Mali) named HSV-2v_7 and classical HSV-2 isolate (from the USA) named HSV-2_USA, GenBank accession number KR135308.

Since HSV-1 and HSV-2 are both prone to recombination (which is seemingly more frequent in HSV-1; Kolb et al. 2013, 2015; Szpara et al. 2014;; Newman et al. 2015), we first performed recombination analyses to identify regions with low recombination rate for further analyses. Although we indeed observed intraspecific recombination events (data not shown), we also identified a minimum of four HSV-1 fragments recombined within HSV-2 genomes. We confirmed these unexpected recombination events by running maximum likelihood (ML) phylogenetic analyses on the recombinant and nonrecombinant regions of the four respective ORF: UL15 (recombinant fragment: 155 bp; positions 33,952–34,106 in the reference genome NC_001798), UL29 (397 bp; positions 60,003–60,399), UL30 (458 bp; positions 66,129–66,586), and UL39 (499 bp; positions 89,393–89,891) (fig. 1 and supplementary fig. S1, Supplementary Material online, p. 6–10). All recombinant fragments included at least one nonsynonymous substitution differentiating HSV-1 and HSV-2 (fig. 2).

A close examination of the alignment actually suggested multiple events in the recombinant regions of UL29 and UL39. Within these regions, several isolates showed apparently shorter HSV-1 inserts (supplementary figs. S2 and S3, Supplementary Material online, p. 11–12). This is compatible with different recombination scenarios, including multiple independent recombination events with HSV-1 or a single initial recombination event with HSV-1 followed by reversal recombination events with HSV-2.

Finally we also observed that, while all published HSV-2 genomes included in our analyses included at least two recombinant fragments (UL29 and UL30) nine HSV-2v sequences were apparently free of any recombinant fragment of HSV-1 origin.

### Phylogenomic Analysis

To further investigate the evolutionary history of HSV-2 and HSV-2v and clarify the context of their recombination events with HSV-1, we first ran phylogenomic analyses in a ML framework and annotated the tree with the recombination status at all four recombinant loci. This resulted in two important observations.

**Fig. 3.**
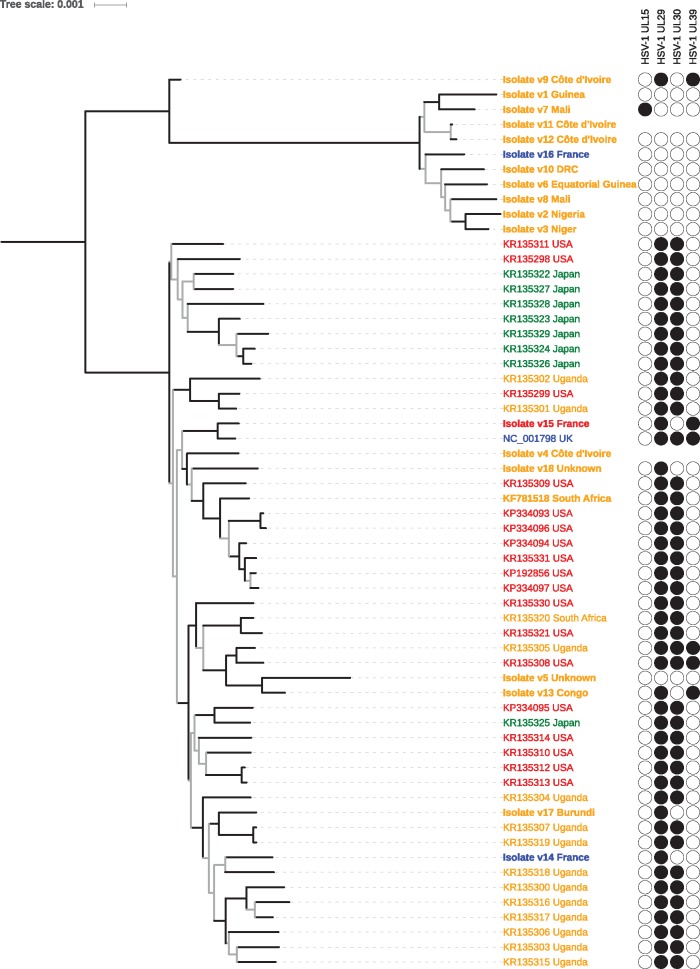
Phylogenomic analysis of HSV-2. This maximum likelihood tree was generated from an alignment of 135,445 positions comprising 60 sequences (including 42 publicly available genomes). Branch leaves are annotated with the accession number and country of origin of the virus; for sequences generated during this study isolate number and country name are in bold. The color code refers to the region of origin: orange for subSaharan Africa, blue for Europe, red for the Americas, and green for Asia. Isolate v15 was obtained from a patient from Martinique (West Indies) and was therefore colored as originating in the Americas. Recombination profiles appear on the right side; filled circles indicate the presence of a HSV-1 fragment, empty circles their absence. Note that (1) for two isolates (v4 and v11) we missed the data at the recombinant loci; (2) given the very short length of their putative recombinant regions we did not mark KR135311, KR135312, and KR135313 as recombinant in UL39. Branch robustness was assessed using Shimodaira-Hasegawa-like approximate likelihood ratio tests (SH-like aLRT); branches supported by SH-like aLRT values < 0.95 are gray. The scale is in substitutions per site. DRC, Democratic Republic of the Congo.

First, we identified two main HSV-2 lineages: a previously unrecognized African lineage only comprising HSV-2v sequences of which most originate from subSaharan Africa; and a worldwide lineage that was also detected in subSaharan Africa and includes some HSV-2v sequences (fig. 3 and supplementary fig. S4, Supplementary Material online, p. 13–14). Additional ML analyses performed on the ten largest nonrecombinant fragments of the alignment showed that members of both lineages have occasionally recombined, as exemplified by the belonging of the HSV-2v isolate v9 (Côte d’Ivoire) to the worldwide lineage for fragments 1, 6, and 7 and to the African lineage for fragments 5, 8, 9, and 10 (v9 was basal to the worldwide lineage for fragment 2, 3, and 4; supplementary fig. S5, Supplementary Material online, p. 15–17).

In addition, the phylogenomic tree supported a very biased distribution of the recombinant fragments, which were almost exclusively found in the worldwide lineage (fig. 3). Although the recombinant fragment in UL15 was only detected in one HSV-2v isolate belonging to the African lineage (v7; Mali), the three others were indeed only found in the worldwide lineage. The recombinant fragments in UL29 and UL30 were nearly ubiquitous in this group, with the only exceptions being HSV-2v isolates. In contrast, the recombinant fragment in UL39 was only found in six sequences, including three HSV-2v isolates.

### Molecular Clock Analysis

To investigate the timing of the interspecific recombination events, we ran Bayesian analyses under strict molecular clock models calibrated by directly using the estimated divergence date of ChHV (Wertheim et al. 2014) or by using the substitution rate derived from this analysis to inform analyses led on an alignment that only included HSV-2 sequences (table 3 and supplementary fig. S6, Supplementary Material online, p. 18–20). Both analyses converged to similar estimates with largely overlapping 95% highest posterior density intervals. This showed that the recombination events in UL29 and UL30 likely affected the ancestor of the worldwide lineage between 34 and 95 thousand years (ky) ago (95% HPD: 29–109 ky). The recombination events in UL39 and UL15 probably occurred later, respectively, within the worldwide lineage <34 ky ago (95% HPD: 29–39 ky) and within the African lineage <15 ky ago (95% HPD: 13–18 ky).

**Table 3:** Times to the Most Recent Common Ancestor of Selected HSV-2 Clades.

	Estimated Date (ky) Median (95% HPD)	
	*Whole genome 2*^a^	*Whole genome 3*^a^
HSV-2	95 (81–109)	107 (90–129)
Worldwide lineage	34 (29–39)	34 (28–40)
African lineage	26 (22–30)	30 (24–36)
HSV-2v7 & closest relative^b^	15 (13–18)	17 (13–20)

a The analyses based on *Whole genome 2* used the divergence with the chimpanzee alpha-herpesvirus 1 as a calibration point; those based on *Whole genome 3* used the evolutionary rate estimate derived from this first analysis.

b The closest relative varied according to the analyses.

## Discussion

Our results show that, contrary to common belief, mixed HSV-1/HSV-2 infections have led to natural recombination events between HSV-1 and HSV-2 (Thiry et al. 2005). These findings are not completely unexpected since such natural interspecies recombination events have already been documented in other mammalian herpesviruses (equine herpesviruses; Pagamjav et al. 2005; Greenwood et al. 2012). What may be more surprising is that recombination within HSV-1 and HSV-2 has often been investigated and detected during the last decade (for a complete review on recombination in alphaherpesviruses, see Loncoman et al. 2016). Yet, the recombinant fragments in UL29 and UL30 went unnoticed, albeit being present in all partial and complete genomes published prior to our study. We note here that early studies focused on recombination used PCR-based approaches and typically covered 2–4% of the genome; to our knowledge, they did not target any of the coding sequences in which we identified recombinant HSV-1 fragments (Bowden et al. 2004; Norberg et al. 2004, 2007). More recently, a number of studies have produced several dozens of HSV-1 and HSV-2 complete (or nearly complete) genomes which were used to examine genome-wide patterns of recombination (Kolb et al. 2011, 2013, 2015; Szpara et al. 2014; Newman et al. 2015). However, it seems that these studies only used single species alignments, which of course prevents the identification of interspecies recombination. Our findings therefore highlight that taxonomically broader analysis of recombination is still warranted, even when studying relatively well-characterized viruses.

A point of particular biological interest is that we detected gene flow from HSV-1 into HSV-2 genomes but none in the opposite direction. Coinfection with HSV-1 and HSV-2 can occur in the anogenital zone on the condition that HSV-1 infection occurs prior to HSV-2 infection; HSV-2 infection seems to protect from further infection of the genitals with HSV-1 (Langenberg et al. 1999; Looker and Garnett 2005). Such coinfections theoretically provide the opportunity of bidirectional gene flow. However, since HSV-1 causes fewer and milder recurrences in the anogenital zone than HSV-2, the transmission of recombinant HSV-1 comprising HSV-2 genome fragments can be expected to be very rare. This alone may explain the apparent unidirectional gene flow from HSV-1 into HSV-2.

Our findings also allow us to speculate on the evolution of HSV-2. The topology of the HSV-2 phylogeny is compatible with an African origin of HSV-2. In addition, the time to the most recent common ancestor of the worldwide lineage (excluding the recombinant HSV-2v isolate v9) is 34 ky (95% HPD: 29-39 ky), which follows the single dispersal event from which all nonAfrican human populations descended (Posth et al. 2016). Therefore, it seems plausible that, following a putative transmission from chimpanzees to the human lineage (Wertheim et al. 2014), HSV-2 diversified in African populations before a single lineage characterized by recombinant HSV-1 fragments (the worldwide lineage) accompanied the migration of *Homo sapiens* out of Africa. African HSV-2 belonging to the worldwide lineage may be the result of later contacts to nonAfrican populations. We note here that this model, and more particularly the notion of an African origin of HSV-2, is also in line with the comparatively high HSV-2 prevalence observed today on the continent (Looker, Magaret, Turner, et al. 2015).

These results may also suggest a link between the recombination and evolutionary histories of HSV-2 since the recombinant fragments detected in UL29 and UL30 are hallmarks of the worldwide lineage. It is tempting to speculate that the success of this lineage was partly due to these early recombination events. The gene products of all four ORF affected by recombination events with HSV-1 are directly involved in the metabolism, replication, or packaging of alphaherpesvirus DNA. The ribonucleotide reductase subunit R1 (encoded by UL39) regulates the pool of cellular dNTP to favor replication, ICP8 (UL29) destabilizes double-stranded DNA to initiate replication, the DNA polymerase (UL30) replicates DNA and the DNA packaging terminase subunit 1 (UL15) is required for proper encapsidation of the viral genome. The recombinant fragments described here may have affected the functions of the respective proteins, as all of them contain nonsynonymous mutations. This notion is still reinforced by hints of multiple recombination events in UL29 and UL39, which could suggest (1) positive selection, assuming multiple independent recombination events with HSV-1, or (2) purifying selection, assuming a single ancestral recombination event has progressively been erased by reversal recombination events. Comparing the biological characteristics of strains from the worldwide and African lineages and/or engineered recombinants thereof may reveal their functional correlates (e.g., in terms of replication and transmission efficiency) and pinpoint the most plausible recombination scenarios at the UL29 and UL39 loci.

Finally, our results highlight that HSV-1/HSV-2 coinfections may have some public health relevance as they already contributed to the genetic diversity of HSV-2 by allowing recombination events with HSV-1. Genomic monitoring will likely be the tool of choice to determine whether interspecific recombination is still an ongoing process and has any clinical implications.

## Materials and Methods

### Sampling and Isolate Preparation

The clinical isolates were selected among 505 HSV-2 isolates recovered from patients experiencing genital herpes hospitalized at La Pitié Salpêtrière-Charles Foix University Hospital. HSV-2v isolates were identified from this collection using a dedicated quantitative PCR system targeting the UL30 gene (Burrel et al. 2015). The presence of the recombinant region within UL30 was further confirmed from tissue samples by Sanger sequencing of PCR products covering the complete coding sequence (Burrel et al. 2015). Because of a shortage of primary clinical sample material, HSV-2 isolates were used in the present study. These isolates had been obtained by propagation in subconfluent monolayers of Vero cells as previously described (Burrel et al. 2010). Only a limited number of passages in cell culture were necessary to generate sufficient viral stocks.

### Molecular Biology

We extracted DNA from 140 µl supernatant using the QIAmp Viral RNA Mini Kit (Qiagen, Hilden, Germany). We quantified total DNA by fluorometry with a Qubit (Thermo Fischer Scientific, Waltham, MA, USA). We also estimated the number of viral copies by quantitative PCR (HSV-2 qPCR), using a newly designed primer set targeting a short fragment of the UL27 gene (HS-2f_B9 5′-CSSCTCSTTCCgMTTCTC and HS-2r 5′-SAYgTgCgTSSCgTTgTA; amplicon length 160 bp) and setting up reactions with the GoTaq qPCR Master Mix (Promega, Fichtburg, WI, USA). HSV-2 qPCR were performed on an AriaMx system (Agilent Technologies, Santa Clara, CA, USA) under the following thermal conditions: 95 °C/5 min followed by 40 cycles of 95 °C/15 s, 58 °C/30 s, and 60 °C/30 s.

We prepared dual-indexed libraries using 1ng DNA extract, the Nextera XT DNA Library Preparation Kit (Illumina, San Diego, CA, USA) and the Nextera XT Index Kit (Illumina). We quantified library molecules using the KAPA Library Quantification Kit (KAPA Biosciences, Wilmington, MA, USA) according to manufacturer’s instructions. We pooled all libraries so individual libraries would all contribute the same number of viral genome copies (using the results of the HSV-2 qPCR).

We then used 200 ng pool DNA to start a double round hybridization capture with a MYbaits Custom Target Enrichment Kit (MYcroarray, Ann Arbor, MI, USA) comprising 9,908 baits (80 bases long) designed to cover the whole genomes of HSV-1 (NC_001806; *Human herpesvirus 1*), HSV-2 (NC_001798; *Human herpesvirus 2*), and varicella zoster virus (VZV, NC_001348; *Human herpesvirus 3*) with a 2× tiling density (note that baits did not cover the whole genomes as low complexity regions were removed from the design). Although we only used representative genomes to generate our bait set, it is now well established that hybridization capture does not require perfect sequence match to function efficiently; for example, viral sequences exhibiting more than 40% divergence to the according baits have already been enriched (Wylie et al. 2015).

For both hybridization capture rounds, we followed manufacturer’s instructions with the exception that only a fourth of the recommended bait quantity was used (Cruz-Davalos et al. 2016). After each capture, we quantified library molecules using the KAPA Library Quantification Kit (KAPA Biosciences). We then amplified capture products using a primer set targeting P5 and P7 and setting up reactions with the KAPA HiFi PCR Mix (KAPA Biosciences). PCR conditions were as follows: 98 °C/2min followed by # cycles of 98 °C/20 s, 65 °C/30 s, 72 °C/45 s, and 72 °C/5min; # was determined taking into the starting number of library molecules, the expected efficiency of the PCR (usually 80%) and the desired amount of amplified capture product (200 ng). We then quantified the PCR product and if the desired amount of DNA was not reached started another cycle of PCR/qPCR. We sequenced the capture product on a MiSeq platform (Illumina) using the MiSeq Reagent Kit v3 (600 cycles; Illumina), generating a total of 9,908,364 reads.

### In Silico Analyses

We removed low quality bases from raw reads using Trimmomatic (LEADING: 30, TRAILING 30, SLIDINGWINDOW: 4:30, MINLEN: 40; Bolger et al. 2014). We then mapped trimmed reads to a HSV-2 reference genome from which we had removed the TRL and TRS regions (NC_001798) using BWA-MEM (Li and Durbin 2009). We finally sorted mapping files and removed duplicates using the SortSam and MarkDuplicates tools from Picard (http://broadinstitute.github.io/picard). Coverage plots were generated with Geneious (Kearse et al. 2012). Coverage was uneven and usually lower in fast evolving regions, e.g., IRL and IRS (supplementary fig. S7, Supplementary Material online, p. 21). We generated consensus sequences using Geneious by calling bases at positions covered by more than 20 reads and for which more than 95% of the reads agreed. We also annotated putative coding sequences with Geneious using a conservative similarity threshold of 60% to coding sequences annotated in the HSV-2 reference genome (NC_001798).

We collected all complete and near complete HSV-2 and HSV-1 genome sequences for which geographic location was known as well as the chimpanzee alpha-1 herpesvirus (JQ360576) and cercopithecine herpesvirus 2 (NC_006560) genome sequences. We discarded 7 HSV-2 (KU310662-8) and 5 HSV-1 (KU310657-61) genome sequences which originated from a single unpublished study and exhibited considerable variation in many coding sequence lengths; the final data set comprised 42 HSV-2 and 42 HSV-1 genome sequences (supplementary table S1, Supplementary Material online, p. 2–5). We also annotated 34 unannotated HSV-2 genomes using Geneious, as abovementioned.

We aligned the extracted consensus and reference sequences using MAFFT v7 (Katoh et al. 2009), resulting in an alignment comprising 104 sequences and 200,070 positions (*Whole genome 1*; see Data Availability). We explored *Whole genome 1* for evidence of recombination using RDP4 (Martin et al. 2015). We set the automated search to use five recombination detection methods (RDP, GENECONV, MaxChi, BootScan, and SiScan) and validated recombination events where >2 methods agreed. We particularly focused on interspecies recombination events, as we initially suspected recombination may have occurred with the chimpanzee alpha-1 herpesvirus (Burrel et al. 2015).

To confirm the detection of recombinant fragments, we extracted the respective ORF and performed ML analyses on the nonrecombinant and recombinant parts of the alignments (*UL15 nonrecombinant*, *UL15 recombinant*, *UL29 nonrecombinant*, *UL29 recombinant*, *UL30 nonrecombinant*, *UL30 recombinant*, *UL39 nonrecombinant*, and *UL39 recombinant*; see Data Availability). For this, we first selected the best model of nucleotide substitution using jModelTest v2.1.4 and the Bayesian information criterion; the selected models were HKY + I+G (*UL15*), F81 + G (*UL15 recombinant*), HKY + G (*UL29*), HKY + G (*UL29 recombinant*), GTR + I+G (*UL30*), HKY + G (*UL30 recombinant*), GTR + I+G (*UL39*), and HKY + G (*UL39 recombinant*; Darriba et al. 2012). We then ran phylogenetic analyses *per se* using PhyML v3 (Guindon et al. 2010). Tree topology, branch lengths and all parameters of the substitution model were optimized. Tree search started from a BioNJ tree and new topologies were generated using an algorithm combining nearest neighbor interchange and subtree pruning and regrafting. Branch robustness was estimated using Shimodaira-Hasegawa approximate likelihood ratio tests (SH-aLRT).

To perform phylogenomic analyses, we discarded HSV-1 and the cercopithecine herpesvirus 2 sequences from *Whole genome 1*, selected conserved blocks therein using Gblocks (Talavera and Castresana 2007), as implemented in SeaView v4 (Gouy et al. 2010), and finally excluded the interspecific recombinant regions as well as the TRL and TRS regions. The resulting alignment included 61 sequences and 135,445 positions (*Whole genome 2*; see Data Availability). We performed phylogenetic analyses on *Whole genome 2* and on a reduced version thereof excluding the chimpanzee alpha-1 herpesvirus (to avoid any long branch attraction effect; *Whole genome 3*) in both ML and Bayesian frameworks. We also performed ML analyses on the ten longest nonrecombinant subalignments that we could identify by running a breakpoint distribution analysis with RDP4 (*Nonrecombinant 1*–*10*, 4,654–12,640 bp; see Data availability). These ML analyses were all performed as described above, using GTR + I+G as a model of nucleotide substitution.

Bayesian Markov chain Monte Carlo analyses were run with BEAST v1.8.2 (Drummond et al. 2012). To determine whether a strict molecular clock model could be applied, we analyzed the clocklikeness of the ML trees using TempEst (Rambaut et al. 2016). The coefficient of variation was <0.03 for the tree derived from *Whole genome 2* and only 0.13 for the tree derived from *Whole genome 3*. We considered these low values as compatible with a strict clock model. We followed the recent suggestion that HSV-2 is itself the result of a cross-species transmission event from the panine lineage and calibrated the clocks by either describing the root age with a normal distribution with mean 1.6 My (million years) and standard deviation 0.1 My (95% confidence interval: 1.4–1.8 My; Wertheim et al. 2014) (*Whole genome 2*); or using the substitution rate derived from the analysis of *Whole genome 2* (7 × 10^−2^ substitutions site^−1^ My^−1^; 95% highest posterior density: 6–8 × 10^−2^ substitutions site^−1^ My^−1^) to inform the clock rate with a normal distribution of mean 7 × 10^−2^ substitutions site^−1^ My^−1^ and standard deviation 7 × 10^−3^ substitutions site^−1^ My^−1^ (95% CI: 6–8 × 10^−2^ substitutions site^−1^ My^−1^). In both cases, the overall model also included a tree shape component, for which we used a coalescent model assuming a constant population size. We used Tracer v1.6 to check that runs had converged and that the posterior was adequately sampled. We then used LogCombiner and TreeAnnotator to combine trees and generate a maximum credibility tree summarizing the posterior tree sample (both softwares are distributed with BEAST). Branch robustness was estimated with their posterior probability. All trees were annotated and prepared for publication using iTOL v3 (Letunic and Bork 2016).

Finally, to control for possible biases induced by our reference-based assembly method, we performed a *de novo* assembly of all HSV-2v genomes with Tadpole 35.82 (written by Brian Bushnell), as implemented in Geneious. Most contigs were relatively short and we therefore had to use reference mapping to further determine longer contiguous consensus sequences. We could identify the same recombinant and nonrecombinant sequences and the trees determined through ML analyses of amended versions of *Whole genome 2* and *Whole genome 3* were very similar to those generated using HSV-2v consensus sequences obtained via reference-based assembly (supplementary fig. S8, Supplementary Material online, p. 22–23; *Whole genome de novo 2* and *3*; see Data availability).

## Data Availability

UL30 sequences have been deposited in GenBank under the accession numbers JX905315-8, KF588390-7, KM068900-1, and KY363355-8. Raw sequences (ERR1380254-71) and genome assemblies (LT797622-36, LT797682, LT797786, and LT7993806) are available at the European Nucleotide Archive under study number PRJEB13741 (http://www.ebi.ac.uk/ena/data/view/PRJEB13741). The following documents are available from the Dryad Digital Repository (http://dx.doi.org/10.5061/dryad.9t21c): (1) alignments: *Whole genome 1*, *2*, and *3*; *Whole genome de novo 2* and *3*; *Non-recombinant 1* to *10*; *UL15 nonrecombinant* and *recombinant*, *UL29 nonrecombinant* and *recombinant*, *UL30 nonrecombinant* and *recombinant*, *UL39 nonrecombinant* and *recombinant*; (2) annotated HSV-2v genomes. All trees generated in this study can be viewed in and exported from iTol (connexion: calvignacs, project: Ancient recombination events between human herpes simplex viruses).

## Supplementary Material

Supplementary data are available at *Molecular Biology and Evolution* online.

## Supplementary Material

Supplementary Data
