# Supplementary material for: Ancient Recombination Events between Human Herpes Simplex Viruses
Source: Mol Biol Evol. 2017 Mar 28;34(7):1713–21. doi: 10.1093/molbev/msx113 (PMC5455963; doi:10.1093/molbev/msx113)

**Supplementary Material**

**Ancient recombination events between human herpes simplex viruses**

Sonia Burrel, David Boutolleau, Diane Ryu, Henri Agut, Kevin Merkel, Fabian H. Leendertz, Sébastien Calvignac-Spencer

**Table S1. Reference sequences used in this study.**

| **Organism** | **Accession** | **Description** | **Country** | **Collection date** |
| --- | --- | --- | --- | --- |
| Cercopithecine herpesvirus 2 | NC 006560 | Cercopithecine herpesvirus 2 |  |  |
| Chimpanzee alpha-1 herpesvirus | JQ360576 | Chimpanzee alpha-1 herpesvirus strain 105640 | USA | 2004 |
| Human herpesvirus 1 | HM585496 | Human herpesvirus 1 strain E06 | Kenya |  |
| Human herpesvirus 1 | HM585497 | Human herpesvirus 1 strain E07 | Kenya |  |
| Human herpesvirus 1 | HM585498 | Human herpesvirus 1 strain E08 | Kenya |  |
| Human herpesvirus 1 | HM585499 | Human herpesvirus 1 strain E10 | Kenya |  |
| Human herpesvirus 1 | HM585500 | Human herpesvirus 1 strain E11 | Kenya |  |
| Human herpesvirus 1 | HM585501 | Human herpesvirus 1 strain E12 | Kenya |  |
| Human herpesvirus 1 | HM585502 | Human herpesvirus 1 strain E13 | Kenya |  |
| Human herpesvirus 1 | HM585503 | Human herpesvirus 1 strain E15 | Kenya |  |
| Human herpesvirus 1 | HM585504 | Human herpesvirus 1 strain E22 | Kenya |  |
| Human herpesvirus 1 | HM585505 | Human herpesvirus 1 strain E23 | Kenya |  |
| Human herpesvirus 1 | HM585506 | Human herpesvirus 1 strain E25 | Kenya |  |
| Human herpesvirus 1 | HM585507 | Human herpesvirus 1 strain E35 | Kenya |  |
| Human herpesvirus 1 | HM585508 | Human herpesvirus 1 strain CR38 | China |  |
| Human herpesvirus 1 | HM585509 | Human herpesvirus 1 strain E03 | Kenya |  |
| Human herpesvirus 1 | HM585510 | Human herpesvirus 1 strain E14 | Kenya |  |
| Human herpesvirus 1 | HM585511 | Human herpesvirus 1 strain E19 | Kenya |  |
| Human herpesvirus 1 | HM585512 | Human herpesvirus 1 strain S23 | Japan |  |
| Human herpesvirus 1 | HM585513 | Human herpesvirus 1 strain S25 | Japan |  |
| Human herpesvirus 1 | HM585514 | Human herpesvirus 1 strain R11 | South Korea |  |
| Human herpesvirus 1 | HM585515 | Human herpesvirus 1 strain R62 | South Korea |  |
| Human herpesvirus 1 | JN400093 | Human herpesvirus 1 strain 134 | USA: Seattle, Washington |  |
| Human herpesvirus 1 | JN420337 | Human herpesvirus 1 strain TFT401 | USA: Seattle, Washington |  |
| Human herpesvirus 1 | JN420338 | Human herpesvirus 1 strain CJ311 | USA: Seattle, Washington |  |
| Human herpesvirus 1 | JN420339 | Human herpesvirus 1 strain CJ360 | USA: Seattle, Washington |  |
| Human herpesvirus 1 | JN420340 | Human herpesvirus 1 strain CJ394 | USA: Seattle, Washington |  |
| Human herpesvirus 1 | JN420341 | Human herpesvirus 1 strain CJ970 | USA: Seattle, Washington |  |
| Human herpesvirus 1 | JN420342 | Human herpesvirus 1 strain OD4 | USA: Seattle, Washington |  |
| Human herpesvirus 1 | JQ730035 | Human herpesvirus 1 strain McKrae | USA | 01-Mar-2012 |
| Human herpesvirus 1 | JX142173 | Human herpesvirus 1 strain Mckrae | USA | 1965 |
| Human herpesvirus 1 | KF498959 | Human herpesvirus 1 isolate RE | USA | 1967 |
| Human herpesvirus 1 | KJ847330 | Human herpesvirus 1 isolate HSV-1/0116209/India/2011 | India | 2011 |
| Human herpesvirus 1 | KM222720 | Human herpesvirus 1 strain MacIntyre | USA | 1951 |
| Human herpesvirus 1 | KM222721 | Human herpesvirus 1 strain KOS | USA | 2010 |
| Human herpesvirus 1 | KM222722 | Human herpesvirus 1 strain KOS | USA | 2010 |
| Human herpesvirus 1 | KM222723 | Human herpesvirus 1 strain KOS | USA | 2010 |
| Human herpesvirus 1 | KM222724 | Human herpesvirus 1 strain F | USA | 2010 |
| Human herpesvirus 1 | KM222725 | Human herpesvirus 1 strain F | USA | 2010 |
| Human herpesvirus 1 | KM222726 | Human herpesvirus 1 strain H166 | USA | 1980 |
| Human herpesvirus 1 | KM222727 | Human herpesvirus 1 strain H166syn | USA | 1980 |
| Human herpesvirus 1 | KT887224 | Human herpesvirus 1 isolate KOS 1.1 | USA | 1964 |
| Human herpesvirus 1 | KT899744 | Human herpesvirus 1 isolate KOS | USA | 1964 |
| Human herpesvirus 1 | NC 001806 | Human herpesvirus 1 strain 17 | United Kingdom: Regional Virus Laboratory, Ruchill Hospital, Glasgow |  |
| Human herpesvirus 2 | KF781518 | Human herpesvirus 2 strain SD90e | South Africa | 1995 |
| Human herpesvirus 2 | KP192856 | Human herpesvirus 2 strain 333 | USA: Texas |  |
| Human herpesvirus 2 | KP334093 | Human herpesvirus 2 strain CtSF-R | USA |  |
| Human herpesvirus 2 | KP334094 | Human herpesvirus 2 strain GSC-56 | USA |  |
| Human herpesvirus 2 | KP334095 | Human herpesvirus 2 strain 1192 | USA: Wisconsin |  |
| Human herpesvirus 2 | KP334096 | Human herpesvirus 2 strain COH 3818 | USA |  |
| Human herpesvirus 2 | KP334097 | Human herpesvirus 2 strain CtSF | USA |  |
| Human herpesvirus 2 | KR135298 | Human herpesvirus 2 isolate HSV-2/US/BID-G19070/8937-1-3336/2003 | USA: WA | 2003 |
| Human herpesvirus 2 | KR135299 | Human herpesvirus 2 isolate HSV-2/UG/BID-G19071/M22987/2007 | Uganda: Rakai | 2007 |
| Human herpesvirus 2 | KR135300 | Human herpesvirus 2 isolate HSV-2/UG/BID-G19072/D30613/2007 | Uganda: Rakai | 2007 |
| Human herpesvirus 2 | KR135301 | Human herpesvirus 2 isolate HSV-2/UG/BID-G19073/F70764/2007 | Uganda: Rakai | 2007 |
| Human herpesvirus 2 | KR135302 | Human herpesvirus 2 isolate HSV-2/UG/BID-G19074/M1119/2007 | Uganda: Rakai | 2007 |
| Human herpesvirus 2 | KR135303 | Human herpesvirus 2 isolate HSV-2/UG/BID-G44423/L22861/2007 | Uganda: Rakai | 2007 |
| Human herpesvirus 2 | KR135304 | Human herpesvirus 2 isolate HSV-2/UG/BID-G19076/H00066/2007 | Uganda: Rakai | 2007 |
| Human herpesvirus 2 | KR135305 | Human herpesvirus 2 isolate HSV-2/UG/BID-G19077/K39924/2007 | Uganda: Rakai | 2007 |
| Human herpesvirus 2 | KR135306 | Human herpesvirus 2 isolate HSV-2/UG/BID-G19078/A76191/2008 | Uganda: Rakai | 2008 |
| Human herpesvirus 2 | KR135307 | Human herpesvirus 2 isolate HSV-2/UG/BID-G19079/J09622/2008 | Uganda: Rakai | 2008 |
| Human herpesvirus 2 | KR135308 | Human herpesvirus 2 isolate HSV-2/US/BID-G19080/44-419851/2007 | USA: MD | 2007 |
| Human herpesvirus 2 | KR135309 | Human herpesvirus 2 isolate HSV-2/US/BID-G19081/44-419851/2007 | USA: MD | 2007 |
| Human herpesvirus 2 | KR135310 | Human herpesvirus 2 isolate HSV-2/US/BID-G19082/44-319857/2007 | USA: MD | 2007 |
| Human herpesvirus 2 | KR135311 | Human herpesvirus 2 isolate HSV-2/US/BID-G19083/10883-2001-13347/2005 | USA: WA | 2005 |
| Human herpesvirus 2 | KR135312 | Human herpesvirus 2 isolate HSV-2/US/BID-G19084/9335-2005-576/2009 | USA: WA | 2009 |
| Human herpesvirus 2 | KR135313 | Human herpesvirus 2 isolate HSV-2/US/BID-G19085/9335-2007-14/2011 | USA: WA | 2011 |
| Human herpesvirus 2 | KR135314 | Human herpesvirus 2 isolate HSV-2/US/BID-G19086/7444-1996-25809/1996 | USA: WA | 1996 |
| Human herpesvirus 2 | KR135315 | Human herpesvirus 2 isolate HSV-2/UG/BID-G19087/J32715/2007 | Uganda: Rakai | 2007 |
| Human herpesvirus 2 | KR135316 | Human herpesvirus 2 isolate HSV-2/UG/BID-G19088/G75809/2007 | Uganda: Rakai | 2007 |
| Human herpesvirus 2 | KR135317 | Human herpesvirus 2 isolate HSV-2/UG/BID-G19089/A76832/2007 | Uganda: Rakai | 2007 |
| Human herpesvirus 2 | KR135318 | Human herpesvirus 2 isolate HSV-2/UG/BID-G19090/D39650/2008 | Uganda: Rakai | 2008 |
| Human herpesvirus 2 | KR135319 | Human herpesvirus 2 isolate HSV-2/UG/BID-G19091/D39765/2008 | Uganda: Rakai | 2008 |
| Human herpesvirus 2 | KR135320 | Human herpesvirus 2 isolate HSV-2/ZA/BID-G19093/SD66/1995 | South Africa | 1995 |
| Human herpesvirus 2 | KR135321 | Human herpesvirus 2 isolate HSV-2/US/BID-G19094/89-390/1989 | USA: MA | 1989 |
| Human herpesvirus 2 | KR135322 | Human herpesvirus 2 isolate HSV-2/JP/BID-G32586/JA1 | Japan |  |
| Human herpesvirus 2 | KR135323 | Human herpesvirus 2 isolate HSV-2/JP/BID-G32587/JA2 | Japan |  |
| Human herpesvirus 2 | KR135324 | Human herpesvirus 2 isolate HSV-2/JP/BID-G32588/JA3 | Japan |  |
| Human herpesvirus 2 | KR135325 | Human herpesvirus 2 isolate HSV-2/JP/BID-G32589/JA5 | Japan |  |
| Human herpesvirus 2 | KR135326 | Human herpesvirus 2 isolate HSV-2/JP/BID-G32590/JA6 | Japan |  |
| Human herpesvirus 2 | KR135327 | Human herpesvirus 2 isolate HSV-2/JP/BID-G32591/JA7 | Japan |  |
| Human herpesvirus 2 | KR135328 | Human herpesvirus 2 isolate HSV-2/JP/BID-G32592/JA8 | Japan |  |
| Human herpesvirus 2 | KR135329 | Human herpesvirus 2 isolate HSV-2/JP/BID-G32593/JA9 | Japan |  |
| Human herpesvirus 2 | KR135330 | Human herpesvirus 2 isolate HSV-2/US/BID-G1929/BethesdaP5 | USA: MD |  |
| Human herpesvirus 2 | KR135331 | Human herpesvirus 2 isolate HSV-2/US/BID-G44422/333-R519 | USA |  |
| Human herpesvirus 2 | NC 001798 | Human herpesvirus 2 strain HG52 | United Kingdom |  |

**Figure S1. Recombination between HSV-2 and HSV-1.**

All panels present maximum likelihood trees. A: UL15 non recombinant segments (nr), B: UL15 recombinant segment (r), C: UL29 nr, D: UL29 r, E: UL30 nr, F: UL30 r, G: UL39 nr, H: UL39 r. HSV-1 labels are in black, HSV-2 in blue and chimpanzee alpha-1 herpesvirus in red. Scales are in substitutions per site. ChHV: chimpanzee alpha-1 herpesvirus.

Figure S1. Recombination between HSV-2 and HSV-1. A: UL15 non recombinant; B: UL15 recombinant.


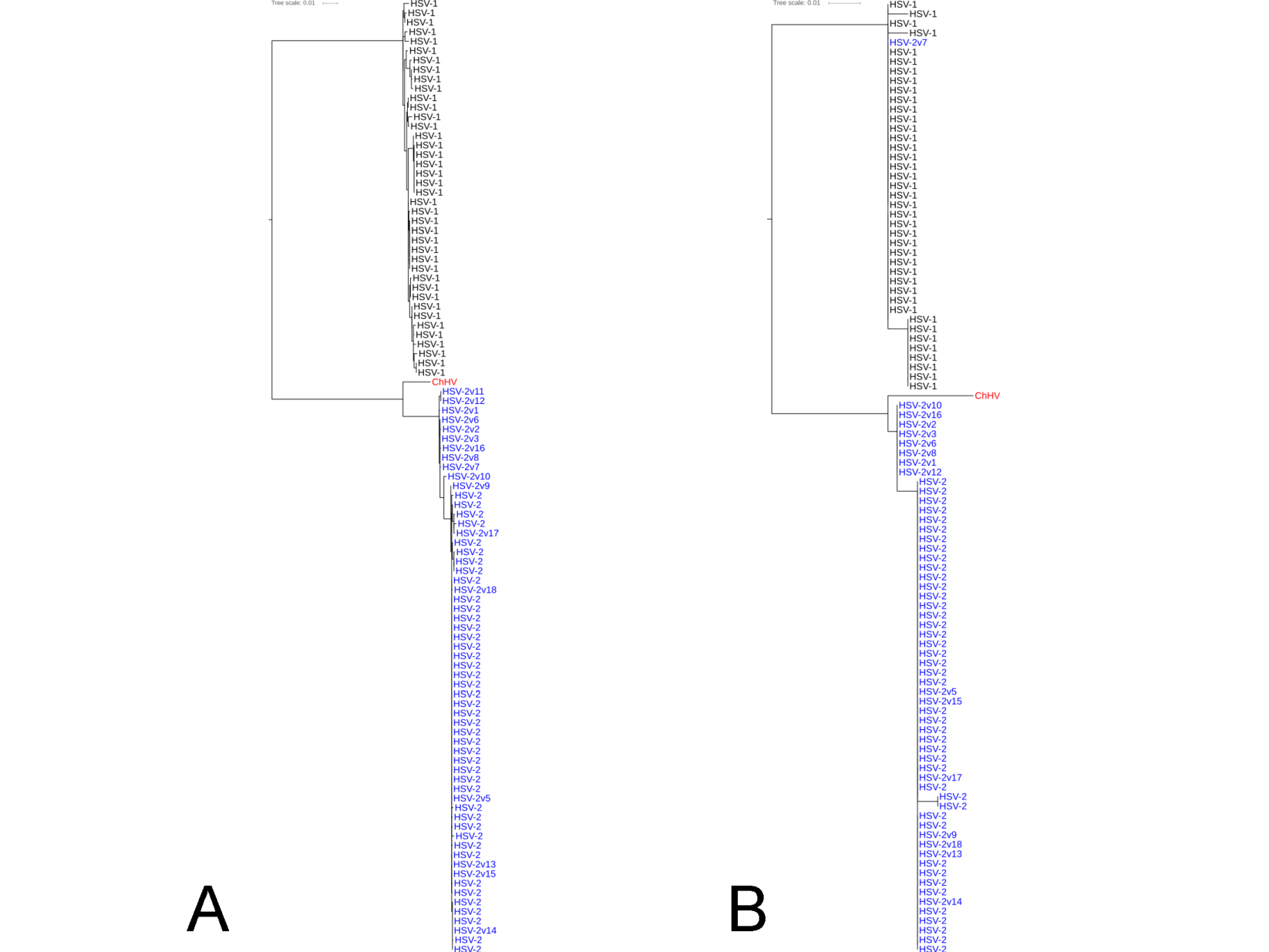


Figure S1. Recombination between HSV-2 and HSV-1 (continued). C: UL29 non recombinant; D: UL29 recombinant.


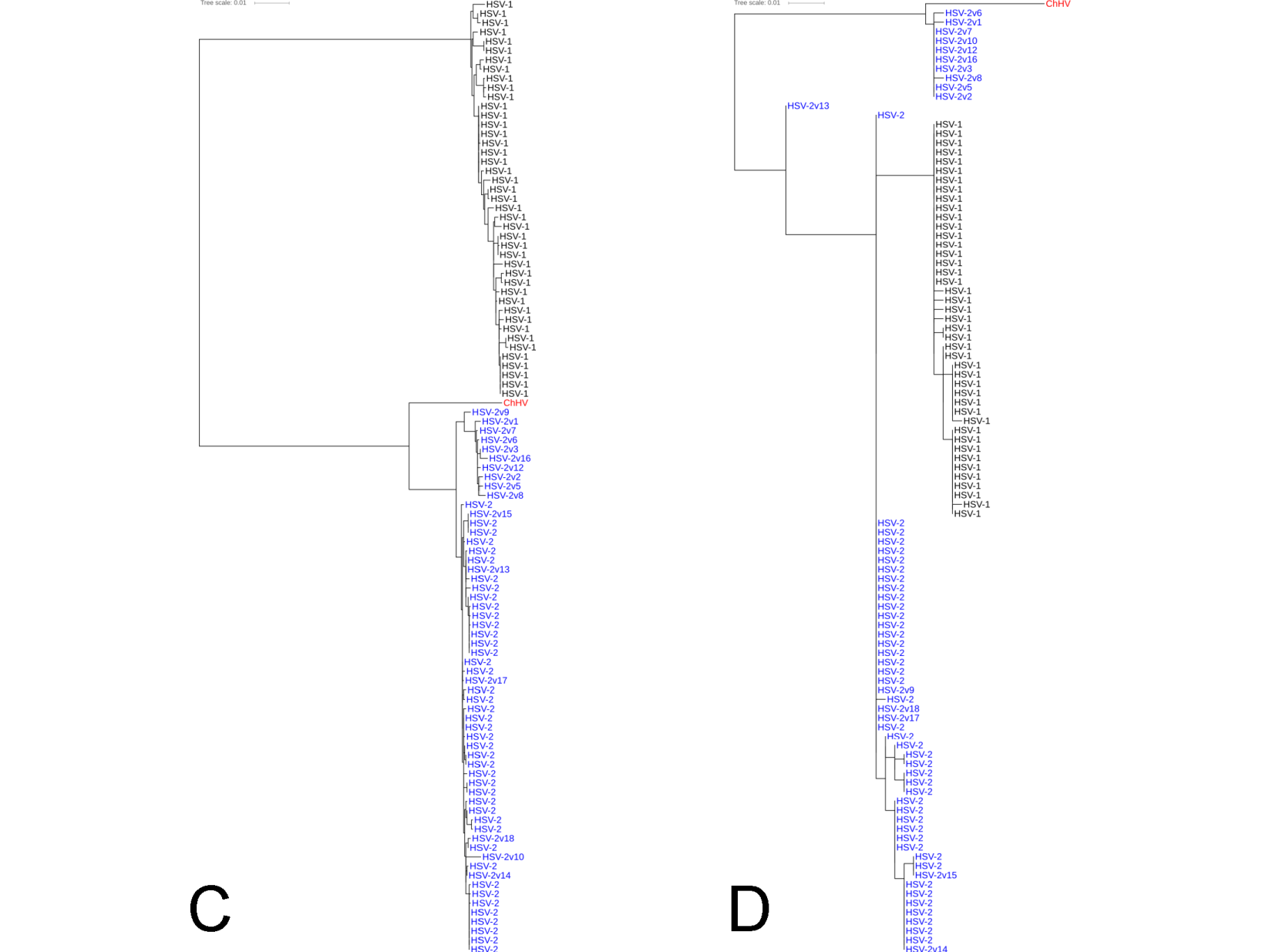


Figure S1. Recombination between HSV-2 and HSV-1 (continued). E: UL30 non recombinant; F: UL30 recombinant.


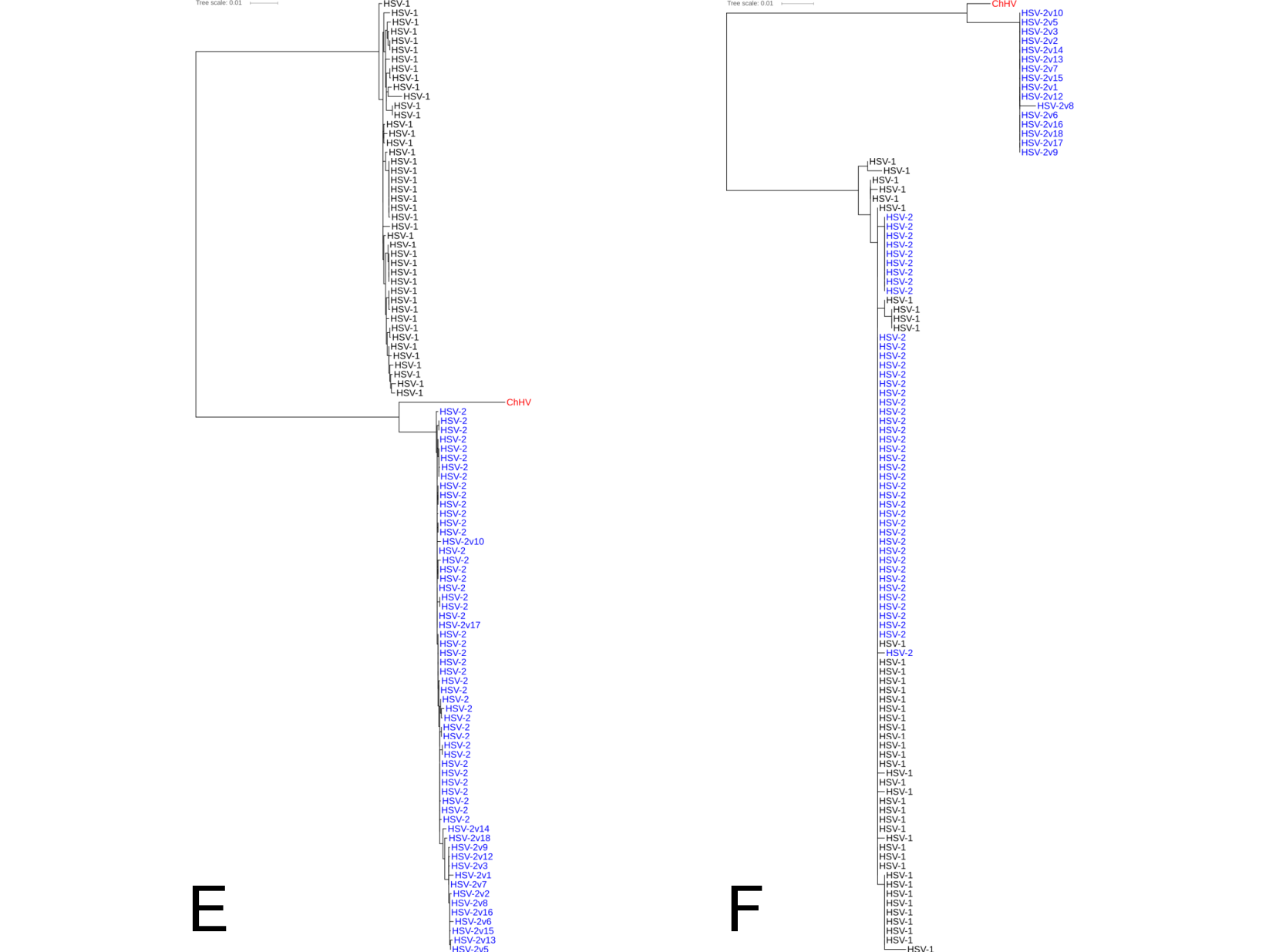


Figure S1. Recombination between HSV-2 and HSV-1 (continued). G: UL39 non recombinant; H: UL39 recombinant.


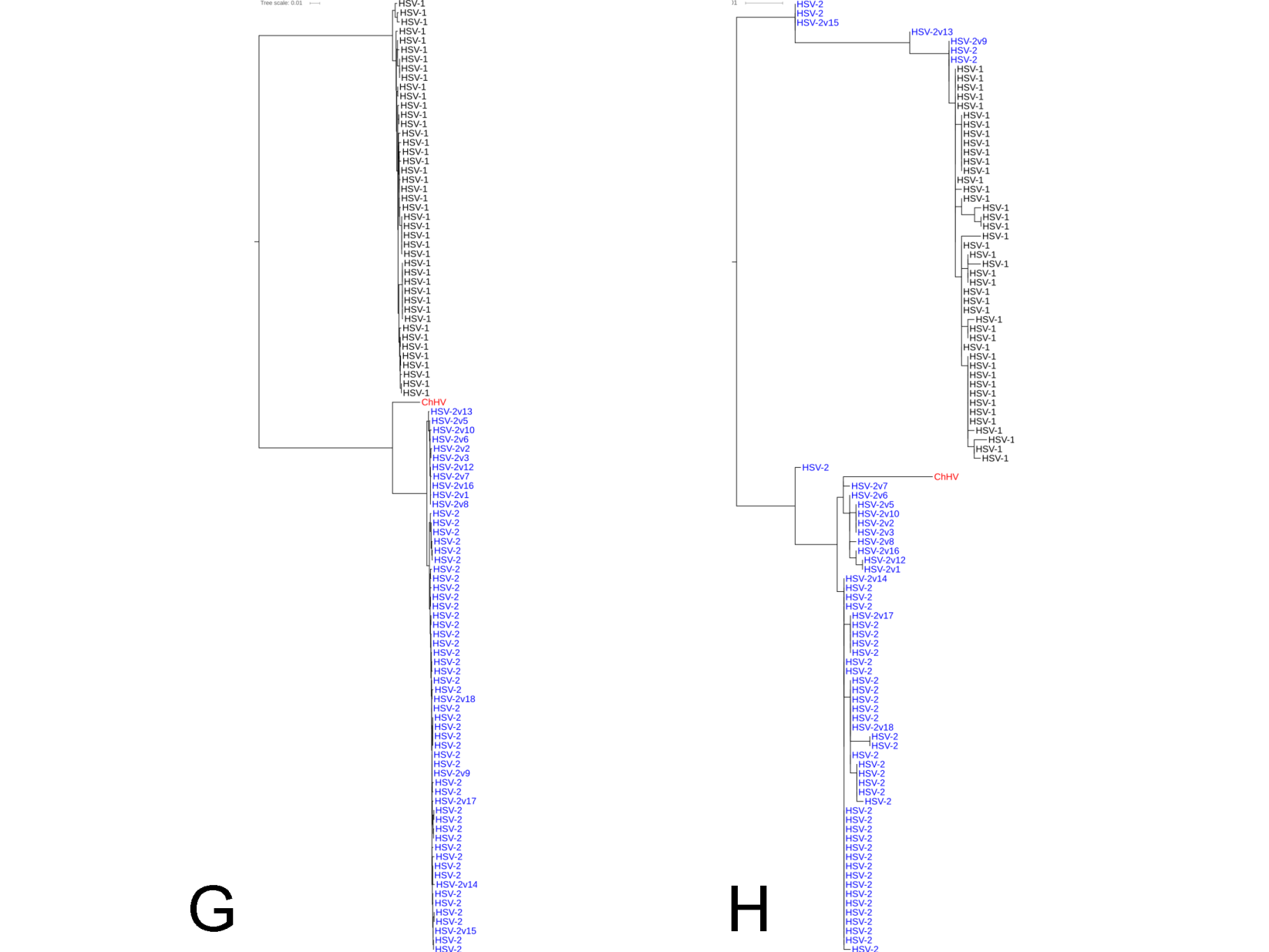


**Figure S2. Alignment of the recombinant region of UL29.**

Zones suggestive of a recombination with HSV-1 are highlighted in blue. A short zone either separating two independent recombination events with HSV-1 or representing a HSV-2 reversal recombination event is pinpointed with a dashed rectangle. Note that HSV-2v_1 and NC_001798 are representatives of 10 and 47 HSV-2 sequences, respectively. This alignment is focused on the recombinant region and therefore only covers parts of UL29 coding sequence.


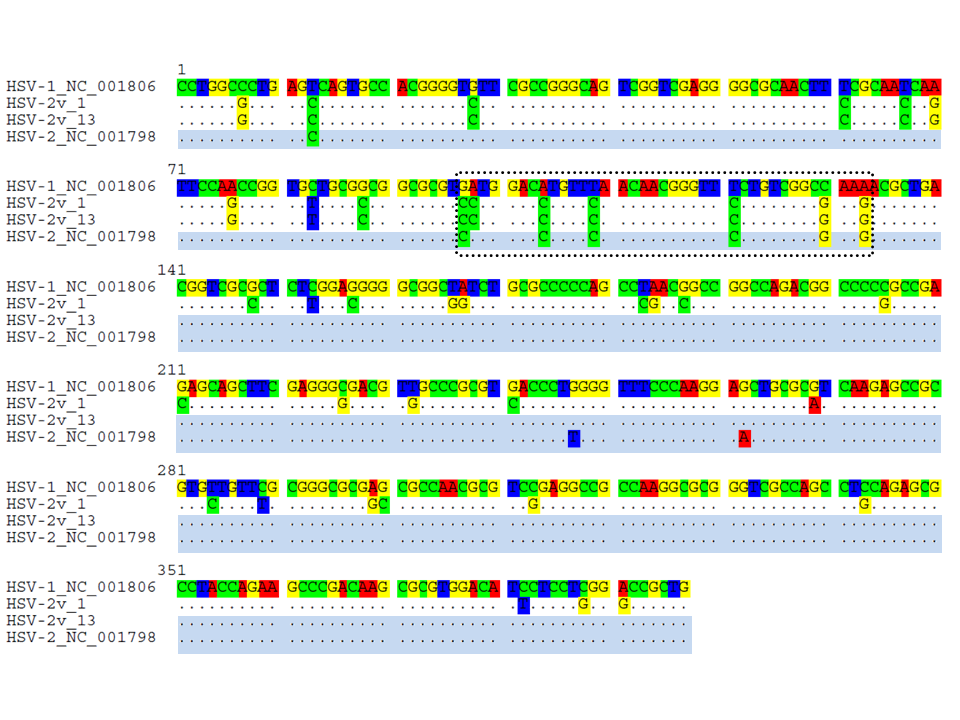


**Figure S3. Alignment of the recombinant region of UL39.**

Zones suggestive of a recombination with HSV-1 are highlighted in blue. Two very short such regions are further highlighted with a dashed line. Many recombination scenarios involving or not reversal recombination events are compatible with this alignment. All recombinant sequences are displayed in this alignment. This alignment is focused on the recombinant region and therefore only covers parts of UL39 coding sequence.


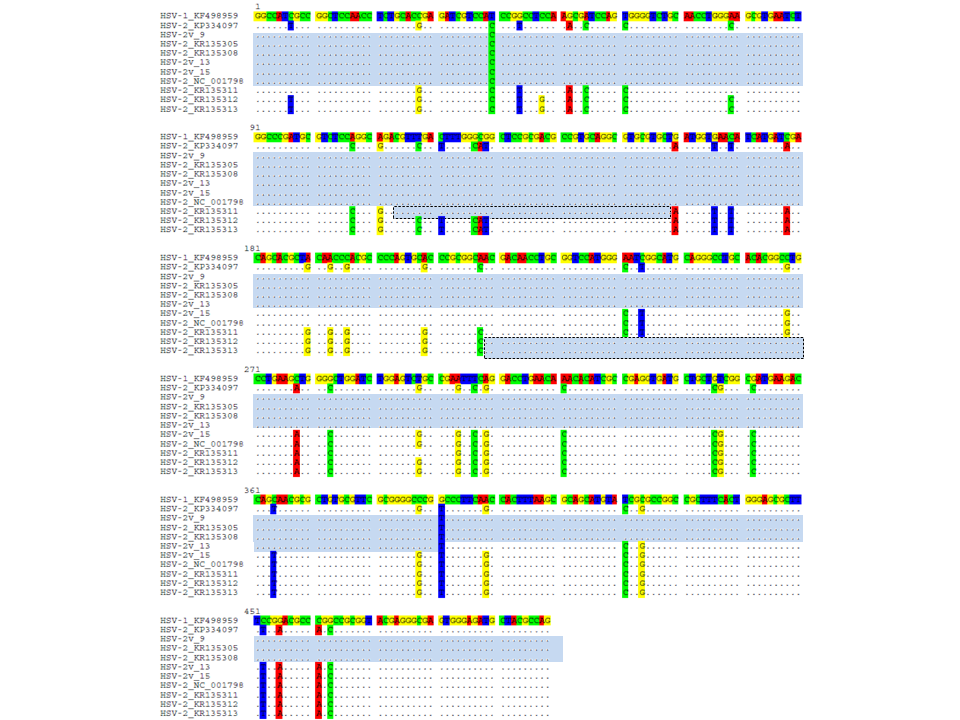


**Figure S4. Phylogenomic analysis of HSV-2 – maximum likelihood.**

This maximum likelihood tree was generated from an alignment of 135,445 positions comprising 61 sequences (including 42 publicly available HSV-2 genomes and the genome of the chimpanzee alpha-1 herpesvirus). Branch leaves are annotated with the accession number and country of origin of the virus; for sequences generated during this study isolate number and country name are in bold. The color code refers to the region of origin: orange for sub-Saharan Africa, blue for Europe, red for the Americas, green for Asia. Isolate v15 was obtained from a patient from Martinique (West Indies) and was therefore colored as originating in the Americas. Branch robustness was assessed using Shimodaira-Hasegawa-like approximate likelihood ratio tests (SH-like aLRT); branches supported by SH-like aLRT values < 0.95 are gray. The scale is in substitutions per site. DRC: Democratic Republic of the Congo.

**
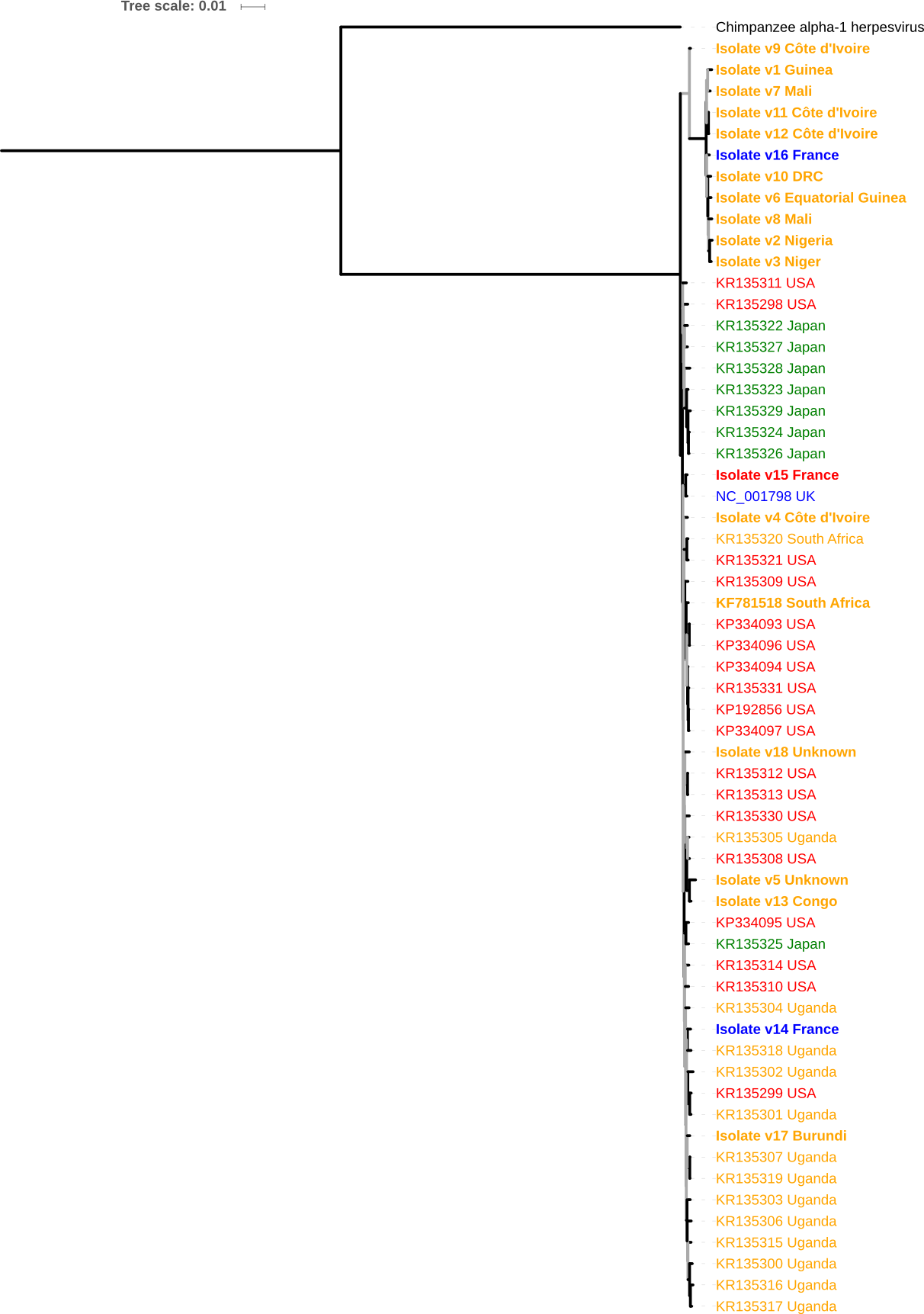
**

**Figure S5. Isolate v9 is a recombinant of members of the worldwide and African lineages – maximum likelihood.**

A: *Fragment 1*, B: *Fragment 5*. In these maximum likelihood phylogenetic trees, tips are annotated with the country of origin of the virus; for sequences generated during this study country names are in bold and are accompanied with an isolate number between brackets, e.g. (isolate v1). The color code refers to the region of origin: orange for sub-Saharan Africa, blue for Europe, red for the Americas, green for Asia. Isolate v15 was obtained from a patient from Martinique (West Indies) and was therefore colored as originating in the Americas. Branch robustness was assessed using Shimodaira-Hasegawa-like approximate likelihood ratio tests (SH-like aLRT); branches supported by SH-like aLRT values < 0.95 are gray. The scale is in substitutions per site. DRC: Democratic Republic of the Congo. DRC: Democratic Republic of the Congo. ML trees from fragments 2-4 and 6-10 can be consulted online on the iTol website (connexion: calvignacs, project: Ancient recombination events between human herpes simplex viruses).

A. *Fragment 1*


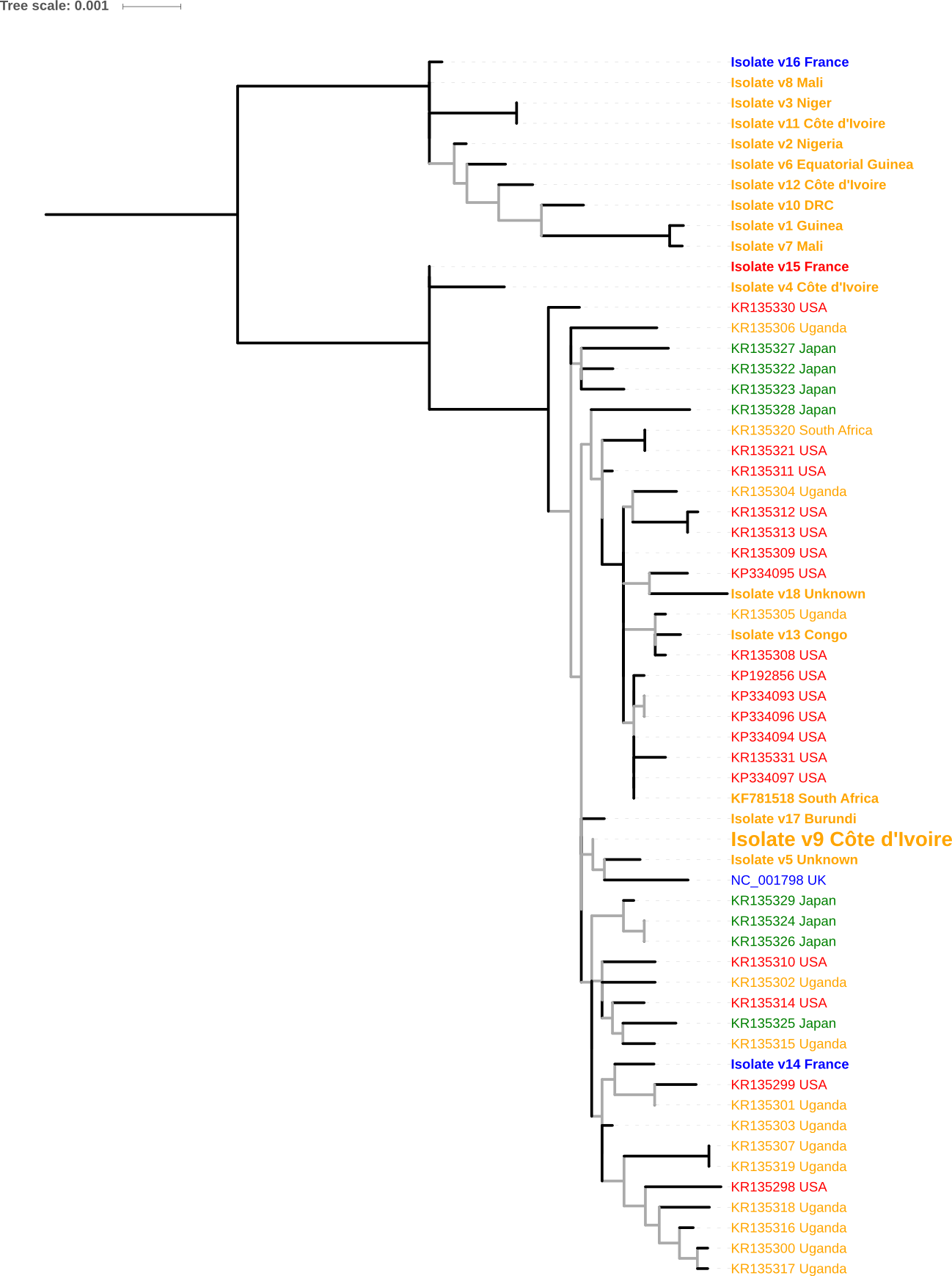


B. *Fragment 5*


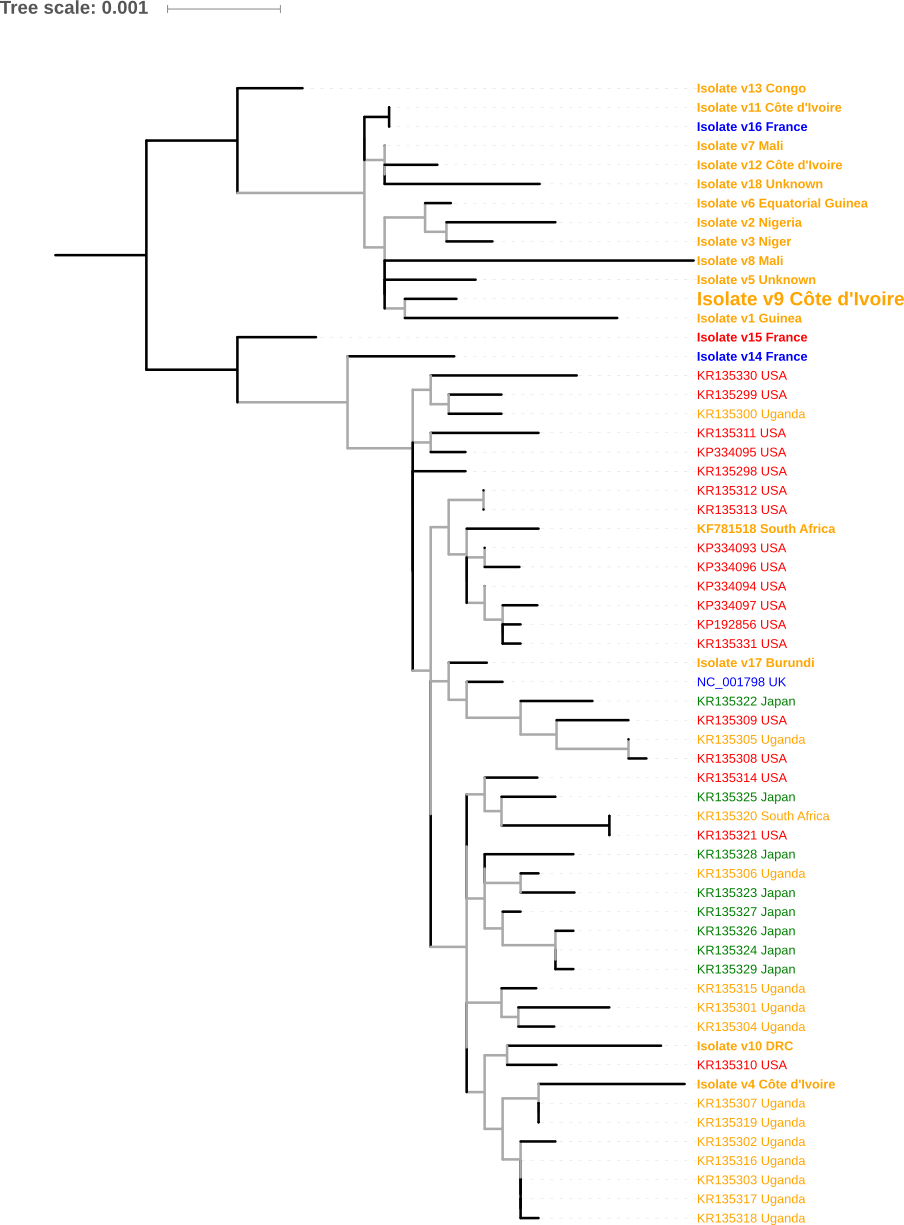


**Figure S6. Phylogenomic analysis of HSV-2 – Bayesian Markov chain Monte Carlo.**

The BMCMC trees in panels **A** and **B** were generated from an alignment of 135,445 positions comprising 61 and 60 sequences (including 42 publicly available HSV-2 genomes and the genome of the chimpanzee alpha-1 herpesvirus). Branch leaves are annotated with the accession number and country of origin of the virus; for sequences generated during this study isolate number and country name are in bold. The color code refers to the region of origin: orange for sub-Saharan Africa, blue for Europe, red for the Americas, green for Asia. Isolate v15 was obtained from a patient from Martinique (West Indies) and was therefore colored as originating in the Americas. Branch robustness was measured by posterior probabilities (pp); branches supported by pp < 0.95 are gray. The scale is in substitutions per site. DRC: Democratic Republic of the Congo.

A. *Whole genome 2*


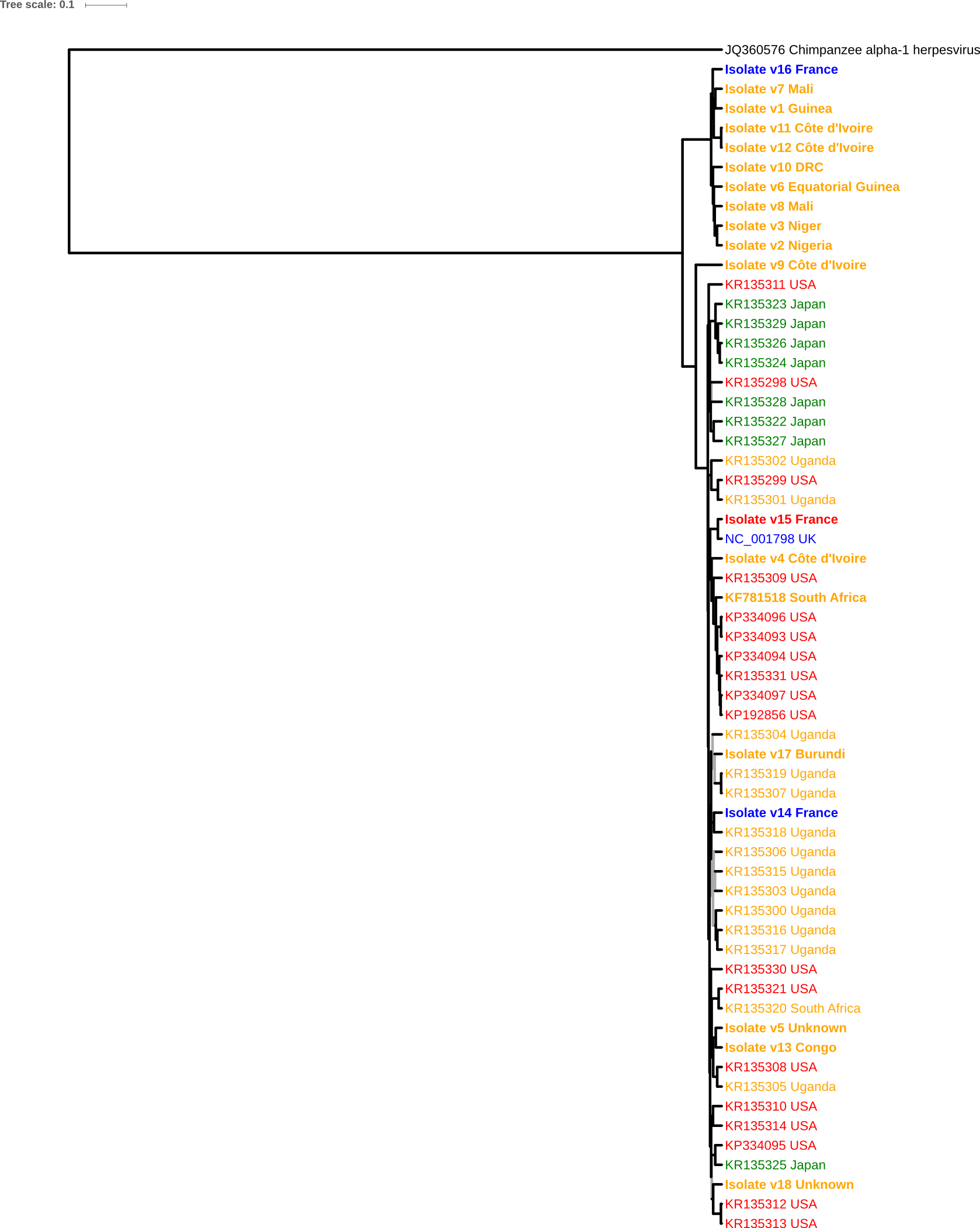


B. *Whole genome 3*


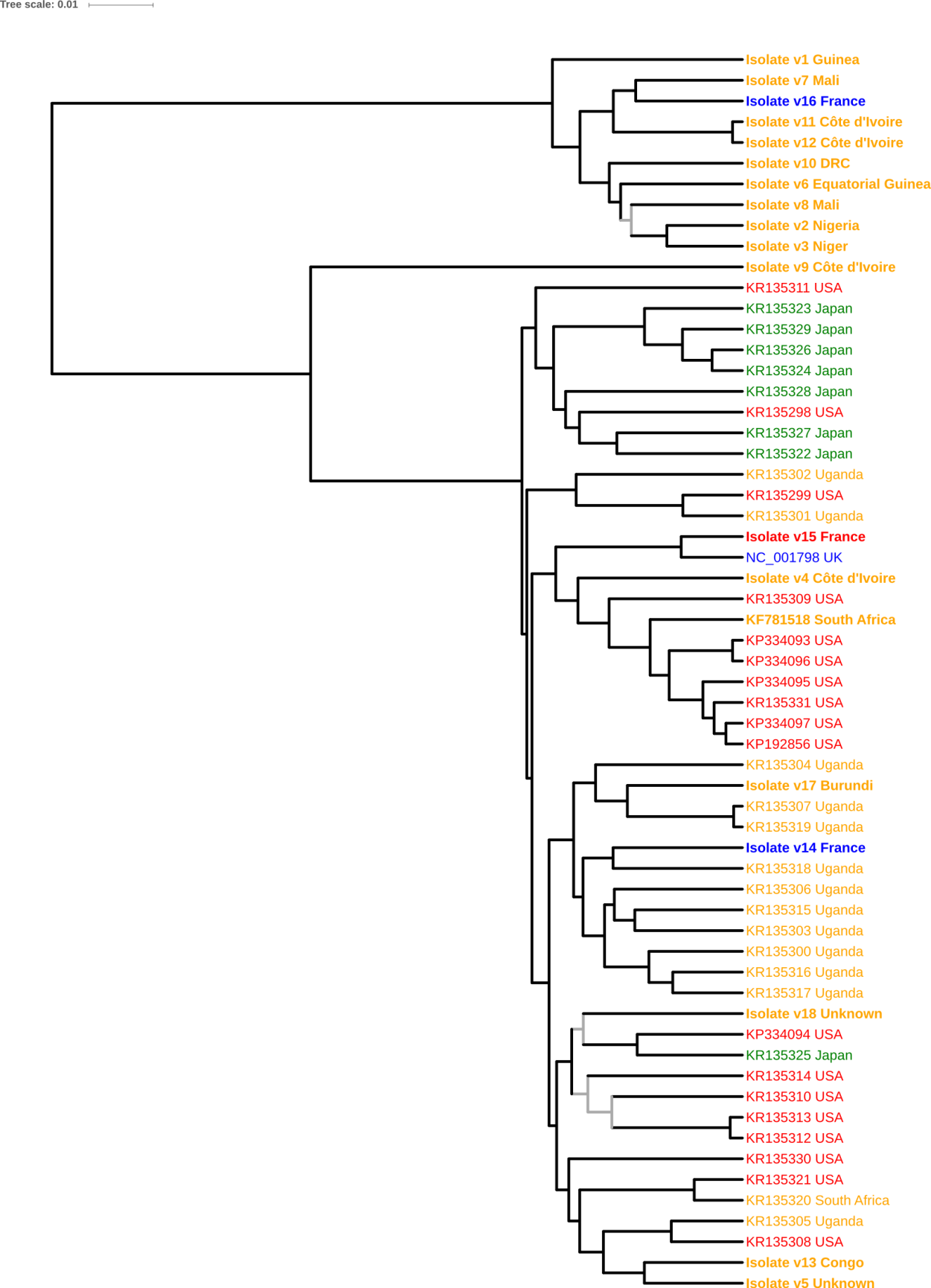


**Figure S7. Examples of coverage plots.**

These two examples correspond to a high (HSV-2v_17) and a low (HSV-2v_4) coverage genome. Positions with coverage above and below 20 reads are highlighted in orange and blue, respectively.

**HSV-2v_17**


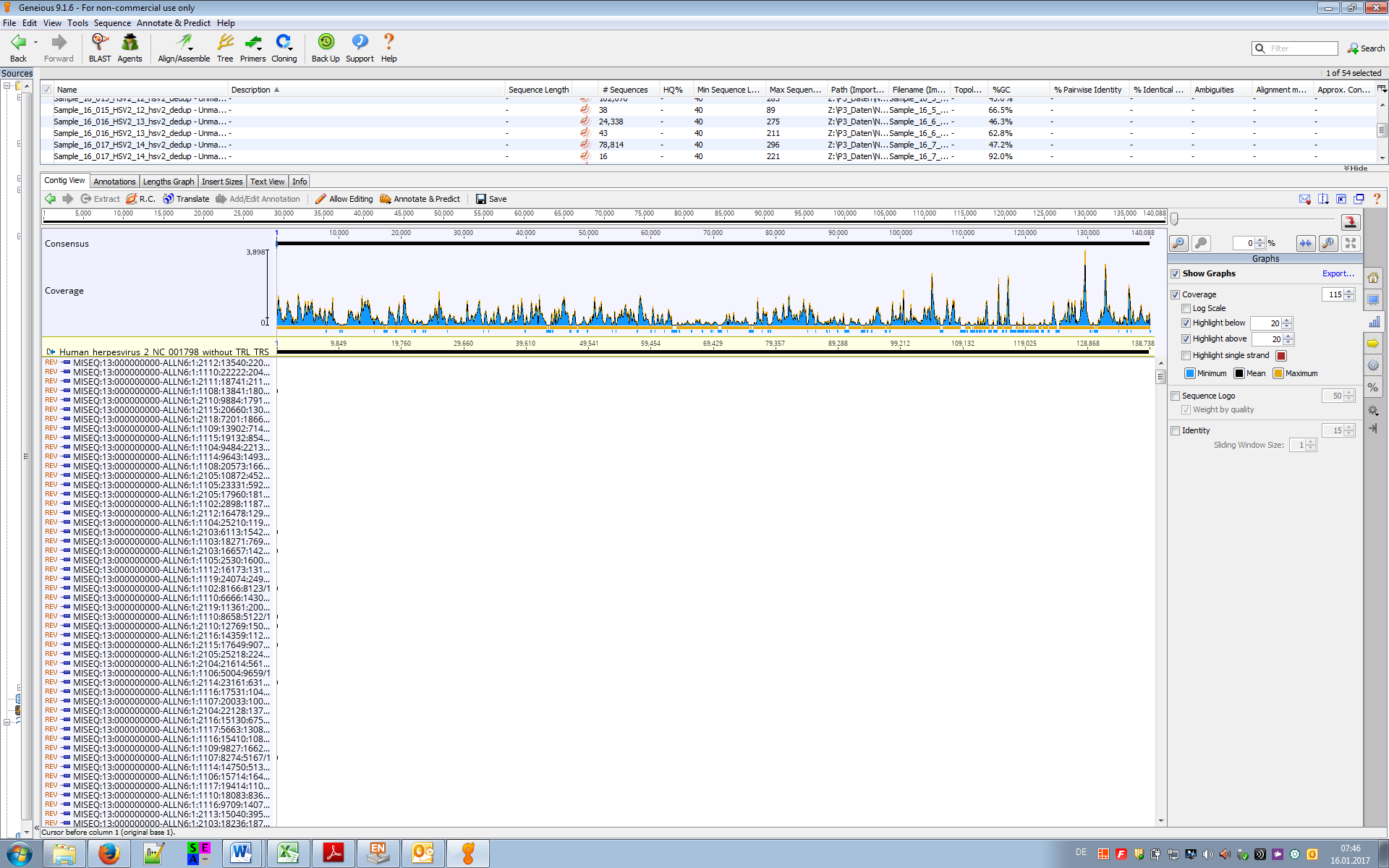


**HSV-2v_4**


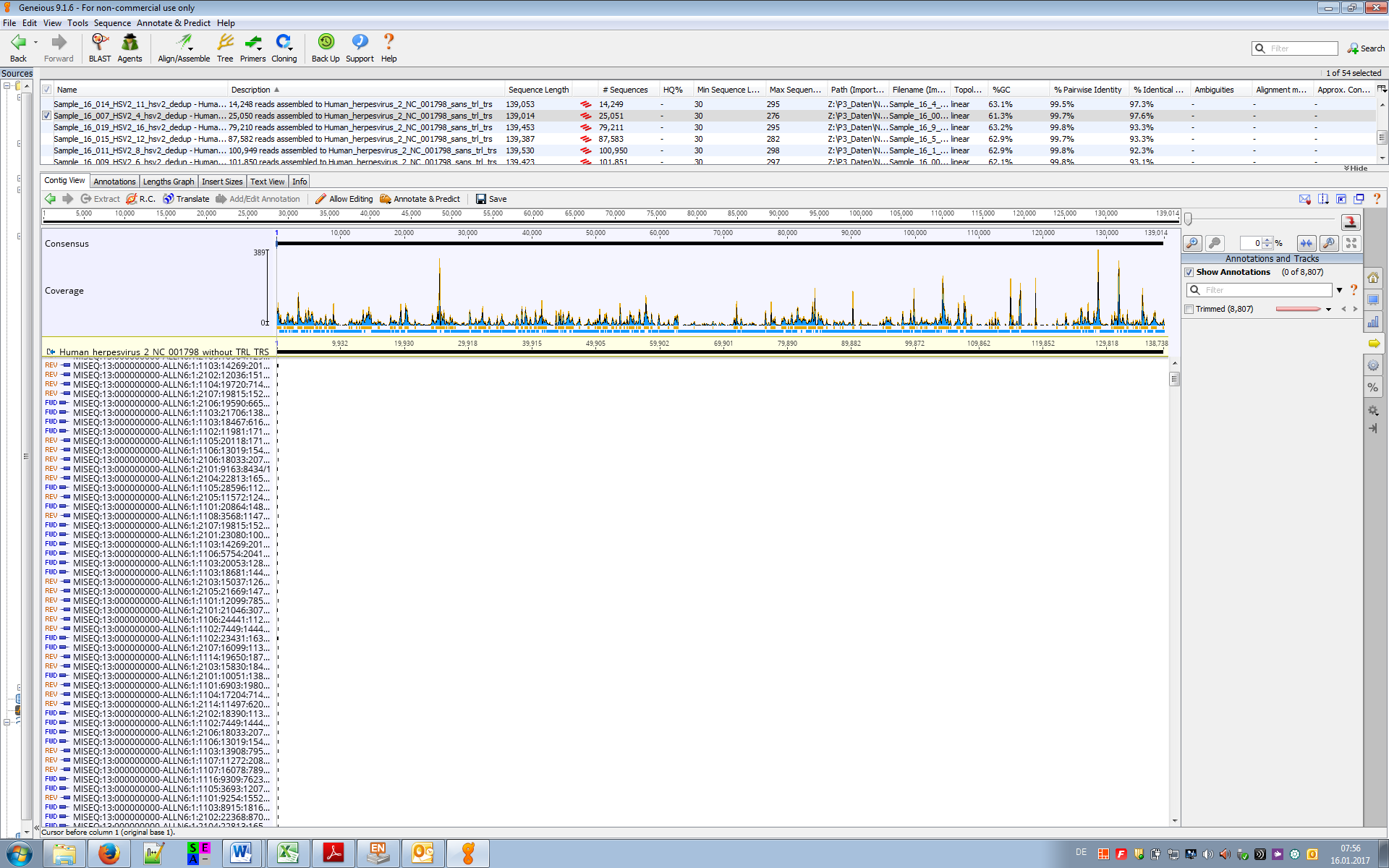


**Figure S8. Phylogenomic analysis of HSV-2 based on de novo contig assemblies of HSV-2v – maximum likelihood.**

This maximum likelihood tree was generated from an alignment of 135,445 positions comprising 60 sequences (including 42 publicly available HSV-2). Branch leaves are annotated with the accession number and country of origin of the virus; for sequences generated during this study isolate number and country name are in bold. The color code refers to the region of origin: orange for sub-Saharan Africa, blue for Europe, red for the Americas, green for Asia. Isolate v15 was obtained from a patient from Martinique (West Indies) and was therefore colored as originating in the Americas. Branch robustness was assessed using Shimodaira-Hasegawa-like approximate likelihood ratio tests (SH-like aLRT); branches supported by SH-like aLRT values < 0.95 are gray. The scale is in substitutions per site. DRC: Democratic Republic of the Congo.


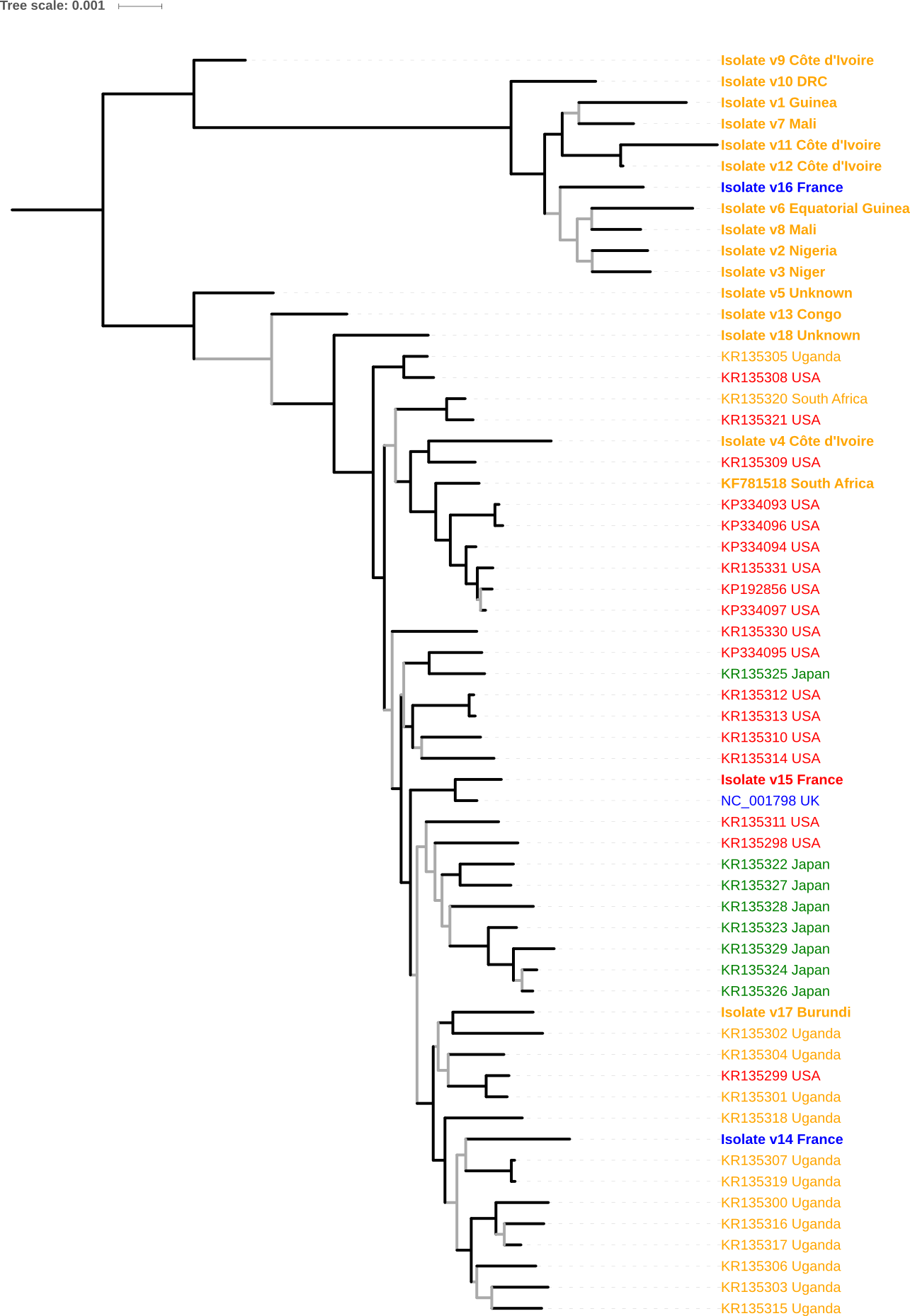

Supplement: Supplementary Data [file msx113_Supp.docx]
